# A Rare Case of Takayasu Arteritis Presenting as Pericarditis with Effusion

**DOI:** 10.1155/2023/6044765

**Published:** 2023-08-24

**Authors:** Ian J. Robertson, David K. Mecham, Lisa M. Conte, Michael F. Loncharich

**Affiliations:** ^1^Department of Internal Medicine, Walter Reed National Military Medical Center, Bethesda, MD 20814, USA; ^2^Department of Rheumatology, Walter Reed National Military Medical Center, Bethesda, MD 20814, USA; ^3^Department of Cardiology, Walter Reed National Military Medical Center, Bethesda, MD 20814, USA

## Abstract

Takayasu arteritis (TAK) is a rare large-vessel vasculitis that is seen primarily in young females of Asian descent and is infrequently diagnosed in the United States. Pericardial effusion with or without pericarditis as a presenting feature of TAK is rare, with only about five percent of cases of pericarditis attributable to any autoimmune etiology. We present a case of a 22-year-old Caucasian woman who presented with a large, symptomatic pericardial effusion of unclear etiology, who after extensive laboratory workup and imaging to include whole-body positron emission tomography (PET) was diagnosed with TAK. In our patient, the use of whole-body PET showing characteristic hypermetabolism within the aortic arch helped secure our diagnosis while avoiding the need for pericardiocentesis. The patient had rapid symptomatic and radiographic improvement with the use of high-dose oral steroids in addition to colchicine and ibuprofen for her pericarditis and associated pericardial effusion. At follow-up just 1 week after initiation of steroids, only trace effusion was identified on transthoracic echocardiogram.

## 1. Introduction

Takayasu arteritis (TAK) is a rare, large-vessel vasculitis that is seen primarily in young females of Asian descent and is infrequently diagnosed in the United States. Early and nonspecific symptoms of fever and night sweats typically occur before the classic symptoms related to claudication, resulting in potentially critical delays in diagnosis and treatment. Furthermore, pericardial effusion with or without pericarditis as presenting features of TAK is rare, with only five percent of cases of pericarditis attributable to any autoimmune etiology [1]. We present a case of a 22-year-old female who presented with a large, symptomatic pericardial effusion as her index manifestation of TAK.

## 2. Case

**Figure 1:**
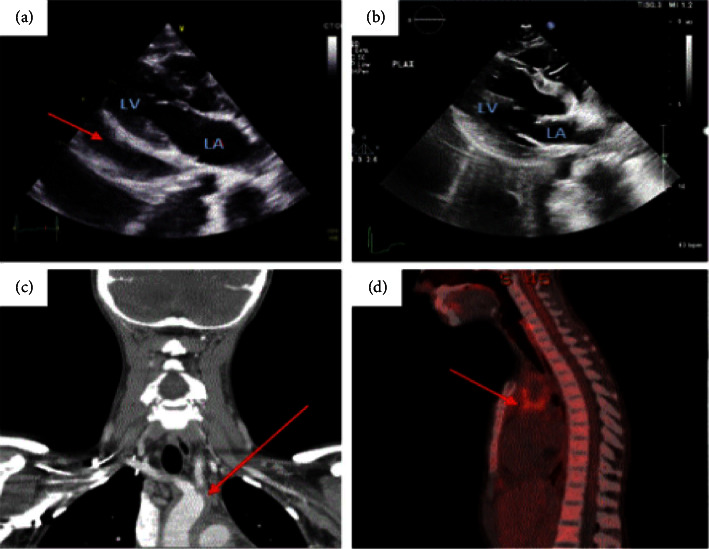
Diagnostic imaging of a patient with Takayasu arteritis. (a) Initial echocardiogram with a large pericardial effusion, red arrow. (b) Repeat echocardiogram without pericardial effusion, obtained one week after starting steroids. (c) CT image of the neck in a coronal plane with thickened proximal aortic and great vessel walls prior to diagnosis, red arrow. (d) PET-CT image in a sagittal plane with active inflammation of the proximal aorta and great vessels, consistent with TAK, red arrow.

A 22-year-old Caucasian female without known chronic conditions presented to our hospital system with five months of intermittent pleuritic chest pain, exertional dyspnea, night sweats, fever, and an approximate 9-kilogram unintentional weight loss. She reported an intermittent globus sensation with meals along with neck and shoulder pain. The patient denied any recent or remote travel outside of the United States, known infectious contacts, and was up to date on age-appropriate vaccinations apart from COVID-19. On examination, it was found that the patient was febrile and tachycardic with muffled heart sounds and decreased breath sounds at the left lung base. The patient did not have asymmetric pulses, carotid bruits, appreciable heart murmur, or blood pressure discrepancy on either arm. Initial laboratory work was initially remarkable only for significantly elevated inflammatory markers (ESR 97 mm/hr and CRP 20.07 mg/dL). Thyroid function panel was within normal limits. Electrocardiogram revealed sinus tachycardia with nonspecific ST wave changes, chest X-ray revealed left greater than right pleural effusion, and a transthoracic echocardiogram demonstrated a circumferential, 3-centimeter pericardial effusion without evidence of tamponade physiology. There was no appreciable valvular abnormality or septal wall thickening noted (Figure 1(a)).

Given these findings, the patient was started on ibuprofen 800 mg every eight hours and colchicine 0.6 mg daily for the treatment of pericarditis with pericardial effusion. However, the underlying etiology of her presentation remained unclear. Subsequent infectious workup included two sets of negative blood cultures, which were tested for both bacterial and fungal pathogens, as well as a negative respiratory viral panel (including COVID-19), acute hepatitis panel which was consistent with immunized status, syphilis, systemic mycoses, strongyloidiasis, human immunodeficiency virus (HIV), and varicella zoster virus (VZV). Evaluation for tuberculosis (TB) with purified protein derivative skin test and QuantiFERON gold assay were also negative. The patient also had no reported history of travel to TB-endemic areas and so concern for active or latent TB in conjunction with negative serologic testing, was deemed exceptionally low by the consulted infectious diseases team. Autoimmune workup included testing for antinuclear antibodies, double-stranded DNA, antineutrophilic cytoplasmic antibody, rheumatoid factor, cryoglobulin panel, and antiphospholipid antibodies which were negative. A computed tomography (CT) of the neck showed abnormal thickening of the aortic arch extending to proximal portions of the brachiocephalic trunk, left carotid, and left subclavian arteries with mediastinal lymphadenopathy (Figure 1(c)). Pericardiocentesis for fluid analysis was strongly considered, however, after a multidisciplinary discussion with rheumatology, infectious diseases, and cardiology regarding differential diagnosis, the decision was made to first pursue additional noninvasive imaging via whole-body positron emission tomography (PET) given the patient remained hemodynamically stable and was symptomatically, improving to assess if the pericardial effusion could be linked to active vasculitis. The study revealed wall thickening of the thoracic aorta, ascending aorta, aortic arch, and proximal descending segments with a standardized uptake value (SUV) max of 5.47 for these vessels (Figure 1(d)).

Radiographic evidence of extracranial large-vessel vasculitis with constitutional symptoms and subtle carotidynia led to a diagnosis of TAK. She was continued on colchicine and ibuprofen for the treatment of her pericarditis. For stabilization of her TAK flare, she was started on oral prednisone 40 mg daily, which was continued for the next month prior to slow taper utilizing a 5 mg decrease every 2 weeks. At follow-up 1 week after initiation of steroids, only trace pericardial effusion was identified (Figure 1(b)) and the patient improved, reporting only mild dyspnea on exertion and intermittent neck pain. Adjunctive oral immunosuppressive therapy was discussed as part of her maintenance regimen. Ultimately, adalimumab was started given family planning concerns. Prior to adalimumab initiation, the patient completed herpes zoster vaccination and was again offered COVID-19 vaccination, which she continued to decline.

At 6-week follow-up, the patient remained stable on prednisone, tapered down to 35 mg, and adalimumab every 2 weeks with normalization of her inflammatory markers. Three months postdischarge, surveillance whole-body PET showed resolution of aortic hypermetabolism, pericardial and pleural effusions, and decreased aortic wall thickness.

## 3. Discussion

Pericarditis with effusion is an exceptionally rare primary manifestation of TAK but should be considered as a possible cause of this clinical syndrome, especially when first-line treatment with anti-inflammatory medication fails, or if symptoms persist or recur. While claudication is a hallmark clinical symptom of a large-vessel vasculitis, particularly TAK, this may only be observed in up to 15% of cases [2]. Furthermore, patients may not exhibit claudication early in their disease course, which can make differentiating TAK from other vasculitides and systemic inflammatory diseases challenging [3].

Imaging can be helpful in identifying early disease and differentiating pericarditis of unclear etiology. In the 2021 large-vessel vasculitis guidelines, the American College of Rheumatology and Vasculitis Foundation recommend noninvasive imaging to include computed tomography angiography (CTA), magnetic resonance angiography (MRA), or positron emission tomography (PET) for assessing potential vessel wall inflammation, without specifying if any of these specific imaging modalities are preferred [3]. PET was used in our case, and can be helpful in atypical presentation of large-vessel vasculitides (LVV), as increased uptake in thoracic aorta and associated branches has been shown to have greater than 95% sensitivity for vasculitis, as compared to about 85% sensitivity for CT or CTA [4]. Regarding MRA vs PET, one prospective study of 84 patients with LVV comparing these two modalities demonstrated greater interrelater agreement within PET for assessment of ongoing disease activity, but MRA was more accurate in assessing disease extent given better detection of both arterial and luminal abnormalities [5]. PET can also be particularly helpful in the differential analysis of pericardial disease through analysis of uptake pattern; in one study of 100 patients with pericarditis of unknown etiology, uptake pattern had a negative predictive value of greater than 94% to exclude malignancy and helped increase yield of targeted biopsies, especially given the often nonspecific conclusions rendered from pericardial biopsy [6]. PET seems to be particularly helpful when potential autoimmune and neoplastic causes must be differentiated from possible infectious etiologies; one larger meta-analysis demonstrated a pooled sensitivity of 87% and specificity of 73% in identifying vascular disease activity in TAK as compared to a group of controls consisting of patients with neoplastic, infectious, and other autoimmune etiologies [7]. Identifying the specific distribution of systemic inflammation through analysis of tissue fluorodeoxyglucose-avidity, as well as potentially abnormal areas of hypermetabolism that may be suggestive of malignancy, allows it to be useful in patients for whom diagnosis is still uncertain after utilization of more conventional imaging methods.

Regarding treatment of TAK, current treatment guidelines recommend initiation of high-dose oral glucocorticoids at 0.5–1 mg/kg/day with an immunosuppressive agent, namely methotrexate, tumor necrosis factor inhibitors or azathioprine, in patients presenting with active TAK [8]. Early and longer-term maintenance treatment with these agents is often required because of the high risk of relapse during steroid tapers or discontinuation of the immunosuppressive agent when clinical remission is achieved [9]. Given a desire to continue breastfeeding, we recommended adalimumab for our patient, which has a demonstrated acceptable safety profile during breastfeeding. Furthermore, adalimumab has a potentially greater initial response rate and lower relapse rate in comparison with other agents such as methotrexate or azathioprine [10, 11].

Ultimately, our case demonstrates an exceptionally rare initial presentation of TAK and the importance of performing a comprehensive evaluation of infectious, autoimmune, and neoplastic etiologies in patients presenting with pericarditis with or without pericardial effusion. It also demonstrates the role for advanced imaging to aid in diagnosis, especially when it allows for the avoidance of a higher morbidity procedure such as pericardiocentesis.

## Data Availability

The data used to support the findings of this study are included within the article.
